# Time Series Expression Analyses Using RNA-seq: A Statistical Approach

**DOI:** 10.1155/2013/203681

**Published:** 2013-03-24

**Authors:** Sunghee Oh, Seongho Song, Gregory Grabowski, Hongyu Zhao, James P. Noonan

**Affiliations:** ^1^Department of Pediatrics, Children's Hospital Medical Center, Cincinnati, OH 45229-3039, USA; ^2^Department of Mathematical Science, University of Cincinnati, OH 45221-0025, USA; ^3^Department of Genetics, Yale School of Medicine, New Haven, CT 06520-8005, USA

## Abstract

RNA-seq is becoming the *de facto* standard approach for transcriptome analysis with ever-reducing cost. It has considerable advantages over conventional technologies (microarrays) because it allows for direct identification and quantification of transcripts. Many time series RNA-seq datasets have been collected to study the dynamic regulations of transcripts. However, statistically rigorous and computationally efficient methods are needed to explore the time-dependent changes of gene expression in biological systems. These methods should explicitly account for the dependencies of expression patterns across time points. Here, we discuss several methods that can be applied to model timecourse RNA-seq data, including statistical evolutionary trajectory index (SETI), autoregressive time-lagged regression (AR(1)), and hidden Markov model (HMM) approaches. We use three real datasets and simulation studies to demonstrate the utility of these dynamic methods in temporal analysis.

## 1. Introduction 

RNA-sequencing (RNA-seq) has fundamentally become the choice of studies of transcriptome [1–6]. From the conventional technologies in microarray and beginning of digital sequencing SAGE [7], a significant hurdle in the analysis of the transcriptome arises from insufficient samples, specifically, in identification of the temporal patterns of gene expression measured at a series of discrete time points. Several data-mining techniques and statistical methodologies have proven to be useful to search temporal gene expression patterns in microarrays [3, 8–34]. Some people have already started to adapt the way we applied in microarrays for RNA-seq data. The main drawback, however, is the loss of discreteness property of read count on transcriptional level, albeit there are no additional advantages in analytical aspects on counts. Given experimental design with sufficient replicate, time points, and sequencing depth [4, 5, 34], attempts to RNA-seq specific methodologies to preserve the elegant count property in time course will contribute to development and application in this area ahead. The last four years witnessed the astonishing publication of statistical methodology studies to identify differential expression between two or amongst multiple groups. Nonetheless most analysis tools remain tied to a static model approach without respect to time, albeit the incisive ultrahigh-throughput sequencing data now provides time series gene expression profile. As the first step towards understanding temporal dynamics in RNA-seq data, temporal analyses often rely on the simple pairwise comparisons [35–47] to infer differentially expressed genes/isoforms at a specific time point versus a reference time point. Differential expression results are then combined to characterize the dynamics over time. Commonly used microarray data analysis methods, such as limma [40], log linear models [39], and ANOVA [26], after variance-stabilizing transformation have also been used for temporal data analysis in RNA-seq as another alternative. However, the very few replications for such data limit the power of these methods. Statistical inference from such high dimensional data structure with the large number of variables and very few observations has presented substantial challenge. More importantly, the pairwise approaches fail to account for the strong temporal dependencies; indeed, higher correlation between neighboring time points is clearly revealed in published gene expression profiles [48] and our real data applications (see Figure 2). Therefore, these pairwise approaches are suboptimal without explicitly modeling the expression dynamics over time nor can the time points that contribute most to time evolution trajectory pattern of a gene's expression be identified. More descriptive methods, such as clustering methods, have also been applied to identify coexpressed gene sets using RNA-seq data [49–51]. Such unsupervised clustering methods implicitly assume that data collected at different time points are independent, ignoring the sequential structure in time series data. It is apparent that potentially useful information on gene regulation and dynamics may be lost with these suboptimal methods, and there is a need to develop statistical methods that can appropriately model and analyze RNA-seq data. We discuss several methods that explicitly model the time-dependent nature of the time series data in this paper. We describe the identification of temporal differential expression (TDE) analyses as well as the ranking of genes to show temporal trajectories with statistical significance. We also discuss the application of time-lagged autoregressive AR(1) models to identify TDE genes as well as hidden Markov models (HMM) to classify different expression patterns by posterior probabilities of latent states. These methods can be applied to study complex factorial designs that interrogate multiple biological conditions simultaneously where multiple time points are studied under two or more biological conditions. Multivariate approaches are presented to identify temporal patterns in coexpressed gene groups and quantify coupled relationship of two distinct trajectories. Here we report an in-depth analysis of temporal patterns based on nonparametric and Bayesian approaches that incorporate the context of inherent time dependence of gene expression *per se*. When these methods are applied for published real datasets, both static and dynamic methods performed well for most temporal genes; however, dynamic methods had particularly a slight edge at low and moderate expression levels. That may be particularly advantageous for years to come for application to data with relatively low signals such as depression and aging data, which on expression compared to tumors in disease tissues. 

## 2. Statistical Methods

### 2.1. Time Series Data Structure

Suppose that a gene expression profile matrix contains *i* = {1,…, *G*} genes and *j* = {1,…, *m*}, *m* different time stages. The *i*th gene expression profile vector, *Y*_*i*_ = [*y*_*i*_(*t*_1_),…,*y*_*i*_(*t*_*m*_)]^*t*^,corresponds to a sequential vector of time points and biological replicates within a time point, namely, where *y*_*i*_(*T* = *t*_*j*_) = [*y*_*it*_*j*_*L*=1_,…, *y*_*it*_*j*_*L*=*l*_] is a vector composed of intraexpression measurements by *L* = *ℓ* biological replicates at time point *T* = *t*_*j*_. We consider a sequence of observations on gene expression profile dataset, made at *m* different time points; accordingly *m* dimensional gene expression vector of gene *i* with observed read counts over time is used hereafter. *y*_*ij*_ = [*y*_*ij*1_, *y*_*ij*2_,…, *y*_*ijc*_]^*τ*^ is *C* = *c* dimensional gene expression vector of gene *i*, time point *j*. The expression profile is a factorial time course experiment and the vector *y*_*ij*_ represents the intraexpression profile of *c* biological condition within a time point. *y*_*ijc*_ = [*y*_*ijc*1_, *y*_*ijc*2_,…, *y*_*ijcl*_]^*τ*^ is an *l* dimensional gene expression vector of gene *i*, time point *j*, *c* biological condition, and *l* different biological individual replicates. If there are not any treated biological conditions, the gene expression time series is simplified in *y*_*ijl*_. 

### 2.2. Statistical Evolutionary Trajectory Index (SETI)

Existing static methods for testing significance of TDE genes in time series RNA-seq data do not consider temporal stochastic ordering dependency property in time, which differs from a typical gene expression profile data, and all static methods assume samples that are distributed independently and are not related to each other instead. However, it is well known that the considerable genes in gene expression profiles related to many developmental biological processes or disease progression are temporally differentially expressed and current expression level is affected by previous one by inherent Markovian property in time series. In the settings of large numbers of variables and with few observation, distribution-free or Bayesian approaches by using useful prior information are more suitable in RNA-seq. To circumvent the limitations and cope with a variety of particular patterns in time course, we present a statistical framework that enables more precise temporal expression profiling by incorporating autocorrelation measurement to determine relationship between consecutive expression profiles. Residuals in one period (or time point) are correlated with those in previous periods (or time point) and ranking individual SETI based on nonparametric regression fit as a gene-by-gene approach. As above, the gene expression level *y*_*ij*_ at *I* = *i*th gene, *J* = *j*th time, *C* = {1,…, *C*} biological condition, and *L* = {1,…, *L*} replicates is fitted by smooth spline regression. The autocorrelations of the residuals are computed by the sliding of all possible cases over the original time series, which are referred to as a trajectory index for given gene. The unbiased estimate of the autocorrelation for each gene is

(1) $$\begin{array}{c} {\overset{\wedge }{\text{ACR}}}_{\text{res},i}\left(k\right)=\frac{1}{\left(m-k\right)\sigma^{2}}\sum_{j=1}^{m-k}\left[Y_{ij}-{\overset{\wedge }{y}}_{i}\right]\left[Y_{ij+k}-{\overset{\wedge }{y}}_{i}\right] \end{array}$$

for any positive integer *k* < *G*. {*y*_1_, *y*_2_,…, *y*_*G*_} is a vector to be contained by *G*-length observations of expression measurement. *P* values for assessing statistical significance are calculated using a permutation test (*N* = 10,000), assuming the absence of temporal differential expression. The confidence interval and trimmed mean of trajectory index are derived by bootstrapping analysis (*B* = 100). The method is based on computing autocorrelations, that is, cross-correlation of gene expression profile across time points to represent temporal pattern. It is applied in a variety of different types of RNA-seq time series data including factorial time course experiments.

### 2.3. Autoregressive Time-Lagged AR(1) Model

We propose to use an autoregressive time-lagged AR(1) model for the identification of temporal and differential gene expression. Hay and Pettitt [54] demonstrated first-order time lag for an application to the control of an infectious disease with count data over time in which the time series observations are examined to identify significant associations with explanatory variables and counts, the incidence of an infectious disease ESBL-producing Klebsiella pneumoniae in an Australian hospital, and the explanatory variable is the number of grams of antibiotic third-generation cephalosporins used over that time period. In order to essentially propose a universal dynamic method with AR(1) model in RNA-seq, we consider models to allow flexibility without covariates in lieu of taking their initial approaches. The details of our AR(1) model for read count gene expression profile over time as a gene-by-gene TDE identification are discussed in the following with mathematical notations. Bayesian framework is defined by (*y*_*ij*_ | *μ*_*ij*_, *i* = 1,…, *n* and *j* = 1,…, *m*) to be independently distributed as Poisson model. We employ their model for RNA-seq read count expression data. 

#### 2.3.1. Poisson Model in AR(1)

From the time series data structure (Section 2.1), we have *m* time points, *c* biological conditions, and *l* replications. Both maize and zebrafish data with single measurements within a time point are applied in this method. 

Consider

(2) $$\begin{array}{c} y_{ij}\sim \text{POI}\left(\mu_{ij}\right),\;\text{where}\;\;i=1,\ldots ,n,\;\;j=1,\ldots ,m,\\ \log\left(\mu_{ij}\right)=w_{ij}+\beta_{i},\\ w_{i1}=\frac{u_{i1}}{\sqrt{1-\varphi_{i}^{2}}},\\ w_{ij}=\varphi_{i}w_{ij-1}+u_{ij},\;j>2. \end{array}$$

And equivalently,

(3) $$\begin{array}{c} \log\left(\mu_{ij}\right)=w_{ij}+\beta_{i},\\ w_{i1}\sim \text{Normal}\left(0,\frac{\sigma^{2}}{\left(1-\varphi_{i}^{2}\right)}\right),\\ w_{ij}\;|\;w_{i,1\ldots ,j-1}\sim \text{Normal}\left(\varphi_{i1}w_{ij-1},\sigma^{2}\right),\;j>2. \end{array}$$

To identify altered gene expression across time series, for each gene, the AR(1) model is applied and inference of *β* is obtained from noninformative priors and time series random effects for sequential expression profile are assumed. This autoregressive model was originally carried out for longitudinal large-scale historical repeated-measurement data. In our study, using the modified assumptions, RNA-seq time series with short time period (4~8 time points) and single observations as gene-by-gene approach are applied to compare the performance of AR(1) model to static methods in identification of differential expression. The posterior probabilities of parameters in the model are estimated through MCMC simulations with *N* = 6,000 iteration and 1,000 burn-in. We provide detailed notations and equations for three dynamic approaches in Supplementary data available online at http://dx.doi.org/10.1155/2013/203681. In the results, we are most interested in autocoefficients to represent time series sequential random effects in the model and we implemented a classification between TDE (temporally differential expression over time) and EE (equally expression over time) set of genes. Similar to statistical differential expression testing for each gene in a classical approach, our implementation of testing in AR(1) model is given by a Bayesian interval estimate, 95% credible interval:

(4) $$\begin{array}{c} H_{0}:\text{if}\;\varphi_{i}=0,\text{EE},\;\;H_{1}:\text{otherwise},\text{TDE}, \end{array}$$

where we consider that gene *i* is temporally differentially expressed (TDE) if the 95% credible interval of *φ*_*i*_ does not include 0; otherwise it is considered to be equally expressed (EE). Also we obtain the tail probability of (*φ*_*i*_ | *y*) of gene *i*, that is, *p*(*φ*_*i*_ > 0 | *y*) or *p*(*φ*_*i*_ < 0 | *y*) for *i* = 1,…, *n* using MCMC. It indicates the significance of differential expression for each gene.

#### 2.3.2. Negative Binomial in AR(1)

A more compelling methodological goal is to infer temporal dynamics when we have replicates within a time point and it is straightforward to establish a negative binomial model with AR(1):

(5) yij~NBC(k,μij), where  i=1,…,n,j=1,…,m,  log⁡(μij)=ωij+βj.

Other parts of the model remain identical as in (3). Here, *y* ~ NBC(*k*, *μ*) means that *y* has its probability function as follows:

(6) p(y;k,μ)=Γ(y+k)Γ(k)Γ(y+1)(kμ+k)k ×(1−kμ+k)y, y=0,1,2,….

This negative binomial distribution has its mean *E*(*y*) = *μ* and its variance *μ* + *μ* ^2^/*k*. The parameter *k* ^−1^ is called the dispersion parameter.

### 2.4. Hidden Markov Model (HMM)

We consider a Bayesian HMM to analyze factorial time course RNA-seq data. Our model follows the seminal work of Yuan and Kendziorski [48] that characterizes all possible temporally differential expression patterns in time series microarray data with two or more biological conditions. Although this early study was encouraging, the HMM was restricted to represent timing differences between biological conditions with binary EE/DE or multiple cases of latent hidden states depending on the number of given conditions at each time point. The extent of temporal changes was not obvious in significantly differentiating between one time point and the next. Taking a HMM approach, we seek SETI and multivariate coupled relationships among distinct trajectories into HMMs in each condition to investigate biological evolutionary trajectory that can be applied to a comprehensive set of RNA-seq time series data to make probabilistic predictions of temporal patterns for how differential expression will occur under different biological conditions. Also, count specific underlying distributions for RNA-seq time series data are used. First, we introduce a mechanism to use the inference of temporally differentially expressed genes in time series RNA-seq gene expression profiles with multiple biological conditions at a given time point. This was achieved by incorporating GP and NBD with corresponding prior information into the HMM for each gene, allowing samples having either multiple replicates or single observations. We investigate properties of the HMM technique such as how it benefits by incorporating hidden variables when making the predictions of temporal patterns of differential expression for given different biological conditions and how the number of chosen latent variables varies with conditions within a stage over a time period. As per Section 2.1, we present how to express hidden states in the given models with subindices composed of *T* time points, *C* different biological conditions (e.g., drug treatments or tissues), and *L* replicates. As RNA-seq experiments generally have small sample sizes, the identification of statistically significant temporally differentially expressed (DE) genes may have limited power. Also, some studies stress the importance of replication in microarray studies, which have inherent variability [4, 5, 33, 34, 55] regardless of how well constructed DE methods are applied. Thus, without replicates, no statistical significance tests are reliable and powerful on detection of TDE. With the reduction in sequencing costs, well-designed balanced RNA-seq experiments with proper sample sizes and time points will facilitate the use of temporal dynamic methods, including AR(1) model. Here HMM is used with samples and 4 biological conditions (different tissues). Consider that the gene expression dataset (*y*_*ijcl*_) has *I* = genes, *J* = time points, *C* = conditions, and *L* = replicates. This algorithm has the Markovian assumption that the expression level at the current time only depends on that at the most recent time. We use hidden states to represent a change in expression levels between different biological conditions. Thus, this framework allows us to detect TDE genes and to facilitate the calculation of the posterior probabilities of all possible TDE patterns. For instance, with three time points, this method can estimate the posterior probability of pattern EE-DE-EE, where EE stands for equally expressed and DE for differentially expressed, respectively. Namely, the main interest is to identify the relationship among the *C* class latent mean values of expression level for each gene g at each time point *T* = *t* denoted by *μ*_*gt*1_, *μ*_*gt*2_,…, *μ*_*gtC*_. Hereby, the primary goal of HHM in time course experiment with multiple different conditions is to infer all potential relationships from different conditions; for simplest case with two biological conditions, it is binary outcome with EE/DE, and for complicated experimental design with more than two biological conditions, suppose that biological conditions correspond to different tissues, hereafter tissues A, B, C, and D. Correspondingly, there are 4 expression profiles *μ*_*gt*A_, *μ*_*gt*B_, *μ*_*gt*C_, and *μ*_*gt*D_, and 15 possible expression pattern states include the following:

(7) $$\begin{array}{c} \text{State}\;1\left[1111\right]:\mu_{1}=\mu_{2}=\mu_{3}=\mu_{4}\\ \text{State}\;2\left[1221\right]:\mu_{1}=\mu_{4}\neq \mu_{2}=\mu_{3}\\ \text{State}\;3\left[1222\right]:\mu_{1}\neq \mu_{2}=\mu_{3}=\mu_{4}\\ \text{State}\;4\left[1121\right]:\mu_{1}=\mu_{2}=\mu_{4}\neq \mu_{3}\\ \text{State}\;5\left[1212\right]:\mu_{1}=\mu_{3}\neq \mu_{2}=\mu_{4}\\ \cdots \\ \text{State}\;14\left[1233\right]:\mu_{1}\neq \mu_{2}\neq \mu_{3}=\mu_{4}\\ \text{State}\;15\left[1234\right]:\mu_{1}\neq \mu_{2}\neq \mu_{3}\neq \mu_{4} \end{array}$$

More generally, the number of all potential patterns as a function of the number of tissues is equal to the Bell exponential number of possible set partitions. Here each state is not observed and needs to be estimated from the data. Therefore, we refer to such states as hidden. For each gene g at each time point *T* = *t*, we want to estimate the probability of each hidden state $p({\vec{g}}_{gt}=k)$ and then we associate an observation model with each state and eventually also compute the most likely sequential states over time to derive timing differences for a given gene g. Fitting a hidden Markov model involves estimating the transition probability matrix *A*, initial probability distribution *π*_0_, and unobserved hidden state at time *T* = *t*, and estimations are done by EM algorithm as described and implemented in the original paper of HMM. The parametric empirical models (PEM) of GP and NBD sample *y* = (*y*_1_, *y*_2_,…, *y*_*N*_) are considered here.

In the GP model, for two biological conditions at each time point and two marginal distributions of hidden states are given the following equations, as shown in Yuan et al., for microarray application. The underlying distributions and joint predictive density (JPD) for discrete count data are incorporated to infer posterior probability distributions:

(8) $$\begin{array}{c} f_{1t}\left(x_{gt}\right)=f_{0t}\left(x_{gt}\;|\;\lambda_{gt}\right)dG_{t}\left(\lambda_{gt}\right) \end{array}$$

under EE state 1 and

(9) f2t(xgt)=∫f0t(xgt1,…,gtn1 ∣ λgt)dGt(λgt) +∫f0t(xgt(n1+1),…,gt(n1+n2) ∣ λgt2)dGt(λgt2)

under TDE state 2.

If *π*_*i*_ represents the proportion of TDE genes at time *t*, then the mixture type of marginal distribution of the data is given by

(10) $$\begin{array}{c} \left(1-\pi_{1}\right)f_{1t}\left(y_{gt}\right)+\pi_{2}f_{2t}\left(y_{gt}\right),\;\text{where}\;\;i=1,\ldots ,d. \end{array}$$

And *f*_*ot*_(*y* | *μ*_*gt*_) = *λ*exp⁡(−*λy*)/*y*!, *x* > 0. *λ*_*t*_ follows a conjugate prior with gamma distribution parameters, shape parameter *α*_*t*_, and rate parameter *β*_*t*_. Thus, three parameters *θ*_*t*_ = (*λ*_*t*_, *α*_*t*_, *β*_*t*_) need to be estimated for a given gene. For the GP model, the Markov chain is assumed to be homogeneous and the marginal distribution of *x*_*gt*_ is the finite mixture ∑_*i*=1_^*d*^ *π*_*i*_ *f*_*it*_. We assume one-step first-order correlation time series structure so that HMM contains with Poisson distributed state-dependent distribution. The goal of this algorithm is to identify a certain set of genes that are TDE in a combination of time series and four different biological conditions, for example, distinct tissue types. To address the utility of HMMs proposed in time course RNA-seq experiments with multiple different tissues, we exploit a parametric hierarchical empirical Bayes model with GP (data w/o replication) and NBD (data w/replications) with beta-prior as a well-modified Bayesian approach [42, 56, 57]. The Newton et al. [57] approach identifies differentially expressed genes for microarray experiment framework in multiple biological conditions at a static time point and similarly Hardcastle and Kelly [42] identify differentially expressed genes either for pairwise comparisons or for multiple group comparisons in an RNA-seq experiment framework at a static time point. For microarray data, Yuan and Kendziorski [48] proposed a HMM for a dynamic time course experiment with multiple conditions Gamma-Gamma (GG) and Log Normal Normal (LNN) to identify genes of interest whose temporal profiles are different across two or more biological conditions. Here, we adapted and extended that approach to a general RNA-seq framework with GP and NBD models as more flexible models. The earlier studies are limited to detect temporal patterns other than ranking/ordering temporal dynamic specific genes during developmental stages, which biologists are more interested in examining. We assume two common underlying distributions for RNA-seq read count. In reality, violation of GP assumptions is very common and in order to account for overdispersion. Alternatively, NBD is applied with a beta-prior. The above inference method provides for continuous trajectory regression involved with timing evolution features to rank temporal genes statistically for a given pattern, as well as such genes' temporal differential expression patterns among conditions. In addition, we examined multivariate identification of temporal expression using the following several metrics.

### 2.5. Coupled Multivariate Identification of SETIs

#### 2.5.1. Granger Causality

The concept of Granger causality between two distinct SETIs assumes that the data at the current time point affect the data at the succeeding time point [58]. To determine Granger causality for each pair of trajectories, we employ standard *F*-statistics to test if the residual values derived from the fitting smoother for gene A are incorporated into the equation for another gene B. If all the coefficients for the measurements of gene B are zero under the null hypothesis, then there is no statistically significant Granger causality between the trajectories for genes A and B. 

#### 2.5.2. Cotrajectory with Glass-d-Score

Similarly, each pair of two trajectories, which correspond to two gene expression levels, is explored by another dependency metric score and detailed notations are described in the following, when there is a given pair of two gene expression profiles:

(11) $$\begin{array}{c} \left(g_{i},g_{j}\right)d_{k}^{ij}=\frac{r_{k}^{ij}-{\overset{-}{r}}_{k}^{i}}{\sigma_{r_{k}^{i}}^{}}, \end{array}$$

where *r*_*k*_^*ij*^ is the correlation coefficient between the expression profiles of (*i*, *j*) among all possible pairs. The null distribution was assumed to have ${\overset{-}{r}}_{k}^{i}(\sigma_{r_{k}^{i}})$ the mean and standard deviation of correlation coefficient between gene *i* and all other genes, respectively.

#### 2.5.3. Correlation Approach

As proposed in Ma et al. and Barker et al. we propose a biologically motivated approach to measure the relationship between two different genes based on their temporal expression profiles in RNA-seq. Ma et al. proposed to consider lagged coexpression analysis to capture the scenario that there is a delayed response of gene B to gene A so that the profile of gene B is correlated with the time delayed profile of gene A. 

### 2.6. Pairwise Methods

In this section, we describe the pairwise methods that we consider in our comparisons with the methods discussed above that can explicitly model the time dependencies nature in the data. For comparisons with our dynamic methods, we examined several popular static methods, including Fisher's exact test for simple two sample comparisons and log linear model for multigroup comparison, which can also be applied for RNA-seq time series data in temporal analysis as intuitive but limited. 

DE analyses: we first employed pairwise condition comparison methods in digital measures at a given static status without respect to time. It is no surprise to take a union set of all possible pairwise comparisons using these static techniques to identify temporal dynamics in relatively small experiments, where single sample for each time point and very few number of time points are contained in experimental design.

(i) Fisher's exact test: from Table 1, the 2-sided *P* value for TDE of each gene is computed with (12) [39]:
  (12) $$\begin{array}{c} \text{Pr}\left(g_{+1,g}=g\;|\;g_{+1.},g_{+2.},\ldots ,g_{.g}\right)=\frac{\left(\begin{array}{c} g_{+1.}\\ g \end{array}\right)\left(\begin{array}{c} g_{+2.}\\ g_{.g}-g \end{array}\right)}{\left(\begin{array}{c} g_{..}\\ g_{.g} \end{array}\right)}. \end{array}$$
(ii) Audic-Claverie statistics.

The Audic-Claverie statistics [59] are based on a distribution *p*(*y* | *x*) over read counts *y* in one sample in one given group informed by the read counts *x* under the null hypothesis that the read counts are generated identically and independently from an unknown Poisson distribution. *p*(*y* | *x*) is computed by infinite mixture of all possible Poisson distributions with mixing proportions equal to the posteriors under the flat prior over *λ*. When the two libraries in a given Solexa/Illumina RNA-seq experiment are of the same size,

(13) $$\begin{array}{c} p\left(y\;|\;x\right)=\frac{1}{2^{x+y+1}}\frac{\left(x+y\right)!}{x!y!}=\frac{1}{2^{x+y+1}}\left(\begin{array}{c} x+y\\ x \end{array}\right). \end{array}$$

These are Audic-Claverie statistics [59] for given read counts *x* and *y*. 

Pooling methods: as with ANOVA in microarray, log linear model and linear models for microarray data (LIMMA), after variance-stabilizing transformation to allow multigroup and multifactor comparisons, can be applied by including a time variable as the main factor in the model [40].

- Log linear model with the Poisson link function (or negative binomial when replicates are available) and likelihood ratio test model. In the model, the time factor, biological condition factors, and their interaction terms are included.
- LIMMA (linear model for microarray) with *F*-statistics under the linear model setting implemented in R package is also applied for time series RNA-seq read count data after variance stabilizing transformation.

Although such static algorithms have demonstrated a successful identification of temporally expressed genes in some degree in the past four years and our study, any temporal dynamic analysis false discovery results in static methods can be introduced due to violation of Markovian assumptions frequently revealed in time series expression profile. As the cost to sequencing continues to decline, there is urgent need for more sophisticated statistical methodologies of power in the identification of temporal expression or for use of characterization of temporal dynamics to assess isoform diversity within a gene level in a future investigation of time series RNA-seq. Ideally, it is very critical to appropriately have a good model to represent observed data since interpretation of a model that does not contain valuable information is useless. For this important purpose, our dynamic methods are compared to these static methods by evaluating the overlap in the number of differentially expressed genes in real data sets. 

## 3. RNA-seq Time Series Data

### 3.1. Three Different Types of Time Series

There are mainly two types of time series in RNA-seq. The first is factorial time series data that include at least two biological conditions to be compared in a given time point and have multiple developmental patterns over time as the number of conditions. The second type of time series has a single condition and corresponding developmental stage. In the third type of time series, there are subsequently two additional subtypes, circadian rhythmic data and cell cycle data. In this study, we formulate the statistical framework of identification of temporal changes in RNA-seq time series for first two types of data and the periodic data-sets are reviewed in “another review manuscript” with discrete Fourier transformation and other methods in a separation in depth.

### 3.2. RNA-seq Real Time Series Datasets

#### 3.2.1. Factorial Time Course Experiment: A Sheep Model for Delayed Bone Healing

We consider this published RNA-seq time series data from a sheep model for delayed bone healing. In Jager et al., surgery was conducted as described in [52, 53] and the newly generated tissues were harvested at different days, 7, 11, 14, and 21 after surgery. For each time point, there are 6 biological replications for both groups except one time point, for day 21 (group I, *n* = 5, group II, *n* = 6), where two groups are defined by standard healing system and delayed healing system. Thus, the authors considered two treatments: standard healing system and treatment with unstable external fixator leading to delayed bone healing. While the standard bone healing system was investigated in a 3 mm tibial osteotomy model stabilized with a medially mounted rigid external fixator, delayed healing was investigated in a 3 mm tibial osteotomy model stabilized with a medially mounted rotationally unstable external fixator. For each treatment, RNA-seq data were collected at 4 time points: 7, 11, 14, and 21 days, with 5-6 individuals' DNA samples pooled together at each time point. In their differential expression, they used the pooled samples from 5~6 lanes of animal samples at one time point and Audic-Claverie statistics were performed using 4 samples over 4 time points by taking a union set of all possible pairwise comparisons using static methods. We reanalyzed their sheep animal time series data using three dynamic methods to identify TDE genes.

#### 3.2.2. Single Transient Time Course Experiment-I

We applied two single biological condition time series data which are interested in exploring developmental transient patterns during a time period rather than timing difference patterns incorporated with multiple conditions at a time as Section 3.2.1 example. Maize leaf transcriptome with four different developmental zones containing two replicates in each time point [50] was employed. This is one representative for time course experiment with single transient expression profile. Tissues were collected from leaf 3 at 9 days after planting 3 hours into the *L* period from four segments: (1) basal (1 cm above the leaf three ligule), (2) transitional (1 cm below the leaf two ligule), (3) maturing (4 cm above the leaf two ligule), and (4) mature (1 cm below the leaf three tip). Thus, maize leaf data with different developmental stages are generated from mRNA isolated from four developmental zones: basal zone, transitional zone, maturing zone, and mature zone. In the differential expression analysis, they simply applied chi-squared static method and *K*-means clustering method that both do not take into account time dependency, but all samples are assumed to be independent. This maize leaf time series data are reanalyzed with proposed methods in this study.

#### 3.2.3. Single Transient Time Course Experiment-II

This is a time series experimental design to be composed of eight stages during early zebrafish development, embryogenesis [51]. In their study, wild-type zebrafish embryos (TLAB) were staged according to standard procedures and about 1,000 embryos were collected per stage (two to four cells, 1,000 cells, dome, shield, bud, 28 hpf, 48 hpf, and 120 hpf) within a tight time window of ~10 min. Their collection of embryos was ensured that all embryos were at the same developmental stage. The identification of long noncoding RNAs (lncRNAs) expressed during zebrafish embryogenesis was explored to assess a diversity of transcripts that are structurally similar to, but noncoding, mRNAs. The analyses of RNA-seq time series expression profiles focused on the identification of temporal dynamics of lncRNAs using the Cuffdiff method in its time series mode with upper quantile normalization, which is also limited to pairwise comparison from previous time point to right next time point. Here, the data reanalyzed the transcriptomic gene expression profile data with 28,520 annotated protein coding genes. To consider the possibility of similarities and differences in comparisons between static and dynamic methods for time series RNA-seq data, we systematically compared both methods with these data. 

### 3.3. Results in Differential Expression Analysis on Static and Dynamic Methods

**Figure 1:**
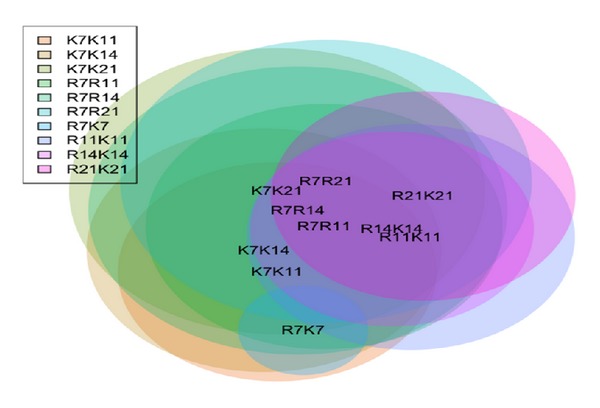
Venn diagram for differentially expressed gene sets detected by Fisher's exact test where the Benjamini-Hochberg FDR is controlled at <0.05. In this figure, as the labels authors used in [52, 53], K represents standard and R represents delayed healing system in a sheep model for two different bone healing systems.

**Figure 2:**
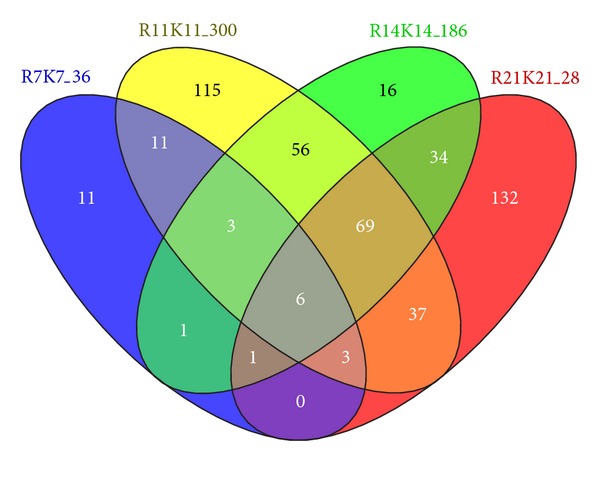
Venn diagram of four DE sets having the number of DE genes between two different healing systems detected by Fisher's exact test with FDR 0.05 at each time point (*t* = 7,11,14, and 21 days). Four time points were compared in simple pairwise comparison between two biological conditions, R (delayed healing) versus K (standard healing system). The label of each set depicts the number of DE genes in the specific comparison. The majority of interaction sets of DE genes between two successive time points implies that high proportion in detected differentially expressed genes at current stage tends to be redetected at next stage revealed by inherent time-dependent structure in time series gene expression profile.

**Figure 3:**
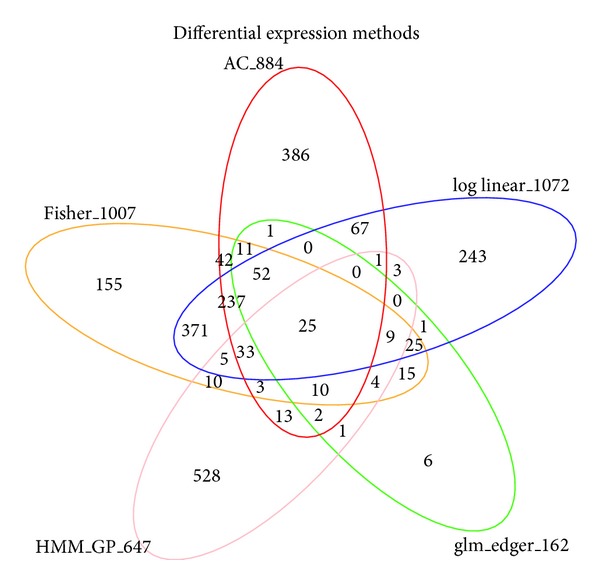
Venn diagram of five DE sets for static and dynamic methods in a sheep model data. In static methods, for two simple pairwise methods, Audic-Claverie statistics and Fisher's exact test were performed and both methods take a union set of all possible pairwise comparisons to identify temporally differentially expressed (TDE) genes across time points and two healing systems. As another static approach, pooling methods of samples, log linear model in [39], and generalized linear model fit in edge R in [36] were performed and detected TDE genes by FDR 0.05. In dynamic HMM method, we identify top candidate TDE genes defined at least showing DE pattern from one time point based on posterior probabilities for latent variables (DE/EE between given biological conditions). On the basis of comparison of the number of DE genes identified by each method, patterns of identification of TDE genes are method specific suggesting validation procedures of methods in biological aspects.

**Figure 4:**
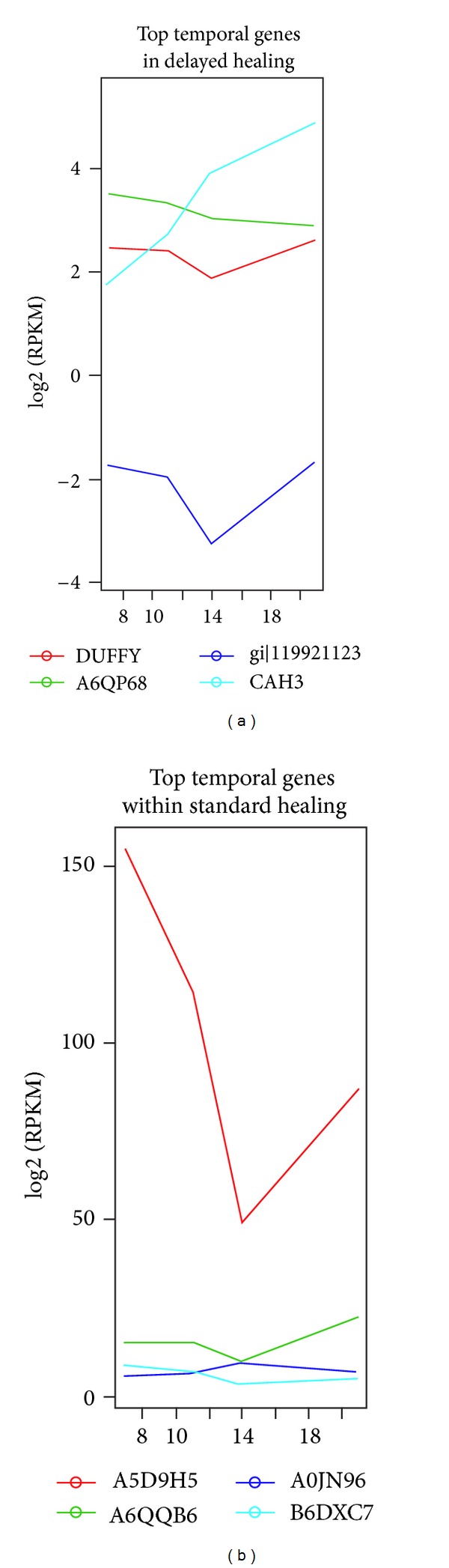
Top candidate temporally differentially expressed genes identified by statistical evolutionary trajectory index (SETI) within each healing system in a sheep model data. Each panel depicts temporal patterns between log2 (normalized expression levels, RPKM) and four different time points under their expression curves. The distinct colors represent significant individual genes ranked by SETI and FDR 0.05 by resampling procedures.

**Figure 5:**
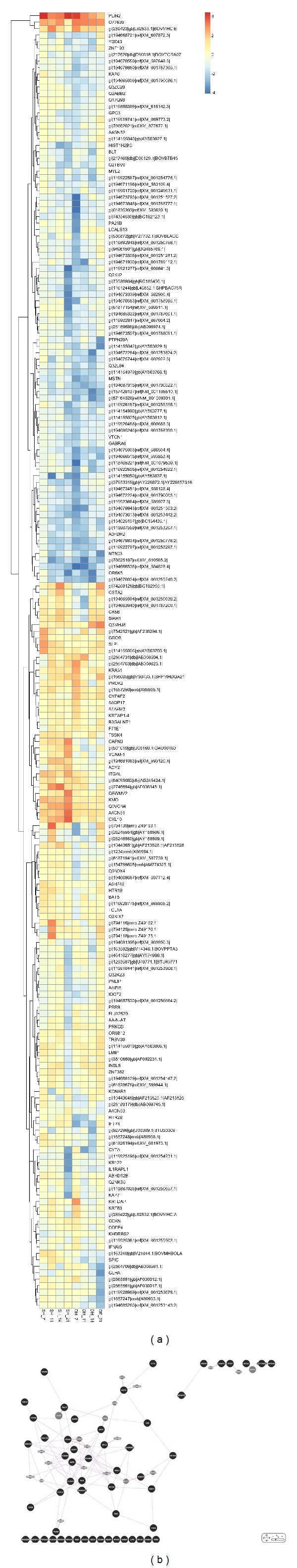
(a) Coexpression patterns from gene clustering in a sheep model data. 200 HMM-specific TDE genes are represented in heatmap. Each row contains a vector of time series expression profile in log2 scale; consequently the visualization in heatmap is originally made up of major three groups, high, moderate, and low expression levels with genens that are not detected by static methods but detected by HMM, of which we selected the most statistically significant 200 genes to present this heatmap. Interestingly, some genes at low expression levels were obviously differentially expressed at log2-scaled FC ~4 up to 5 and even some genes that significantly show temporal patterns at high expression levels were also detected, yet those genes were not detected by existing static methods suggesting that HMM method reassuringly has higher sensitivity and robustness than other existing static methods in identification of differential expression regardless of expression levels. (b) Gene functional pathway and network analysis with 528 HMM specific TDE genes in a sheep model data. To explore biological functions in this gene set further, whether or not those are genuinely differential expression or random noise by chance in terms of biological insights, gene ontology (GO) and KEGG pathway analysis were performed to identify meaningful functionalities and some meaningful functions related to developmental process (intermediate mesoderm formation, regulation of cell growth involved in regulation of muscle adaptation, intermediate mesoderm formation, etc.) and gender specific terms (granulosa cell development and maternal placenta development) are detected as we anticipated to confirm the sensitivity of dynamic HMM method. The purple and pink legends represent coexpression and physical interactions across genes, respectively, and black nodes are query genes in networks.

For the sheep data, the authors applied the Audic-Claverie method to the normalized expression values, RPKM, to compare later time points to the reference time point (7 day) in both groups. After all pairwise comparisons, they combined the sets of differentially expressed gene sets with 884 genes detected in total from 24,325 mappable genes. Based on these 884 genes, they performed hierarchical clustering to identify gene clusters. Each cluster was then subject to gene ontology analysis to find significant biological functions. The differential analysis performed in original paper is based on static differential analysis method. We reanalyzed their sheep factorial time course experiment data to identify TDE genes over time through dynamic methods, HMM, SETI, and AR(1) model to account for correlated time-dependency structure. HMM identifies temporal patterns with classification of DE/EE at each time point by posterior probabilities, whereas SETI with statistical significance from permutation resampling procedures and AR(1) model with gamma Poisson Bayesian assumption on count data are applied within single biological condition, separately. Results obtained by these dynamic methods compared those of static methods, simple pairwise methods, Audic-Claverie statistics and Fisher's exact test, and pooling static methods, glmFit in edgeR, LIMMA, and log linear model as shown Figure 3. To identify temporal dynamics by assuming correlated data structure, we performed HMM modeling with Poisson-gamma since there were no replicates. AR(1) model and SETI significance tests were also done within each biological condition. Temporally differential expression gene sets detected by these dynamic methods were compared with the results of simple pairwise tests and pooling methods. From the HMM, 646 temporal dynamics of DE calls are identified to represent DE in at least one time point. The HMM model only explores different temporal patterns of DE/EE states and does not rank the genes by statistical significance, but is classifying gene expression profile into a number of temporal patterns by posterior distribution of latent states. Because of this limitation, we employed the SETI and AR(1) models to discover developmental transient patterns in each condition. The trimmed mean time evolution trajectory index is presented for the top three candidate temporal genes in each bone healing system. The 95% confidence interval of bootstrapping and FDR of permutation re-sampling are shown in Figure 4 and Table 2. To determine temporal dynamics and meaningful biological functions, only HMM-specific TDE genes which are not contained in static methods are further explored in gene clustering and biological functional network analysis as shown in Figures 5(a) and 5(b), respectively. In the results, they obviously showed temporally differential expression implying that loss of information to assumption of stochastic time-dependent structure might lead to false discoveries and less power of detection. To discover temporal transient patterns of differential gene expression within each biological condition; healing system, we performed SETI and AR(1) model approaches for each condition, SETI results are given in Figure 4 showing top candidate TDE genes, of which some genes such as gi*|*119921123 and B6DXC7 are of low expression levels which we were not able to detect in static methods. In the second data for our study, we have reanalyzed maize leaf transcriptome data to identify TDE genes with static and dynamic methods and compare between two. In their paper, they investigated leaf development gradient in time series gene expression data at successive stages (4 time points: base, tip: basal, transitional, and maturing) and identified a gradient of gene expression from base to tip: basal (23,354) > transitional (22,663) > maturing (22,036) > mature (21,332) from a total of 25,800 annotated genes. In the differential analysis in times series RNA-seq data, they used the method proposed in Marioni et al. [40] for pairwise analysis. A total of 16,502 genes were found to be differentially expressed in at least one of the comparisons. They then performed *K*-means clustering and showed eighteen clusters along the four developmental zones (Base, −1 cm, 4 cm, Tip). To compare gene sets detected by our dynamic methods with their gene lists, dynamic methods, SETI, and AR(1) model are applied again in this study and all temporally differentially expressed genes are presented in Supplementary Tables 1 and 3, where filtered gene set to be tested in differential expression has 5273 and 12,322 temporal dynamic transcripts from 42399 transcripts through SETI and AR(1) model, respectively. On the basis of significant temporal expression, we compared dynamic methods to static methods, which were used in the original paper without accounting for correlated data structure type. As the third real data application, to identify temporal dynamics, we have reanalyzed the third data, zebrafish embryonic transcriptome, focusing specifically on the identification and characterization of temporally differential expression using statistical evolutionary trajectory index and autoregressive time-lagged AR(1) model. We furthermore implemented both methods to rank temporal genes by statistical significance. As consequence of the resampling-based procedures and posterior probabilities of autocorrelation, it was possible for gene-by-gene approach to order temporal genes by two dynamic methods and identify genes associated with cotemporal dynamics. To investigate such paired temporal dynamics, we examined the relationships between genes using bivariate identification methods. Glass-s-d score is reported in Supplementary Table 5. Likewise, the statistical evolutionary trajectory index with statistical significance for zebrafish data is given in Supplementary Table 2, where we filtered out genes by coefficient of variation (CV) criteria remaining 12,034 genes. Overall, both methods show more robustness at low and moderate expression levels when compared to existing parametric static methods indicating that our methods achieve relative improvements in test of identification of temporal genes and AR(1) model shows more sensitive TDE calls than SETI resampling procedure in two real data applications. Here, we examined how different results are obtained by dynamic time series methods. For simple pairwise static methods, we employed Audic-Claverie statistics and Fisher's exact test as these two methods have been widely used in previous studies. They showed highly concordant results on other RNA-seq datasets compared to DEGseq, DESeq, edgeR, and baySeq (data not shown). In differential analysis with simple pairwise methods, we took a union set after all pairwise comparisons across a time period and amongst different biological conditions as these methods only consider two pairwise comparison testing and confirm the results to those of original papers. For pooling static methods, LIMMA, log linear model, and edgeR R package with glmFit are carried out to identify TDE genes. To compare with above static methods, we employed three dynamic methods described in the previous sections. The results are shown in Figures 1 and 3. Figure 2 shows how dependent structure is observed in patterns identified across time points, 36(23), 300(277), and 186(134), of the previous TDE gene set, genes in 64% ~ 92% percentage are differentially reidentified at the right next time point, implying that there is temporal dependent structure in sheep healing system RNA-seq time series data. 

### 3.4. Bivariate Dynamic Analysis for RNA-seq Time Series

In systems developmental biology where characterization of complexity of various time course data likely leads to address inference of temporal dynamic patterns from transcriptome, we are not often really interested in exactly how only a single gene is temporally differentially expressed at a particular time point or period. This knowledge would neither answer an understanding of how biological networks in temporal dynamics of gene regulation work nor enable predicting any cooperative sets of genes to occur under biological conditions across time points. Thus, it is well known that genes work collaboratively together in a structured biological network; these biological phenomena underscore the importance taking into account the multivariate techniques when modeling temporal dynamic gene expression. Since it is not known beforehand which gene features are connected to each other, investigators sought to define informative relationships between individual gene patterns to identify many relevant classes of dynamic temporal gene expression patterns. We explored highly correlated relationships between temporal gene sets detected by bivariate dynamic methods. Pairs of trajectories were further investigated to explore the coupled coordinated relationships between different temporal patterns based on the three dependency metrics in Section 2.5. Significance levels of such relationships were estimated by bootstrapping resampling. The methodologies to test any coupled relationship to pairs of district gene expressions are based on (1) Granger causality, (2) correlation-basis approach, and (3) Glass-s-d score as defined in [60]. In order to efficiently identify copaired temporal dynamics, using zebrafish data, we first identified statistically significant TDE genes and ran Glass-d-score based on gene permutations.  Figure 8 demonstrates coupled temporal dynamics with log-scaled expression level.

### 3.5. Gene Functional Pathway and Network Analysis

**Figure 6:**
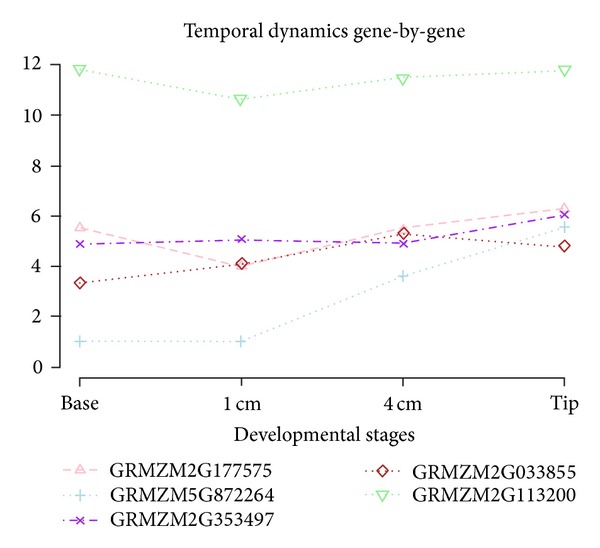
Gene expression curves for five significant TDE genes in maize leaf data. Mean expression curves are presented from two replicates during four developmental stages comparing temporal patterns each other in identifying statistically significant trajectories.

Once temporal dynamics in gene-by-gene test and in gene-to-gene interaction were determined, the resultant temporal gene expression sets detected by ranking individual analysis and multivariate approaches, respectively, were further explored to reveal temporal relationships underlying biological processes based on gene ontology and functional network/pathway analysis. Sheep gene symbols with 21,865 genes were converted into human gene symbols with 15,343 using BioMart in R package [61]. In gene ontology analysis through Avadis NGS [62], 528 specific genes which were detected by uniquely HMM were further analyzed. Interestingly, as 63 females were sampled in the data, some of gender-specific GO terminologies among significant ontology terms were identified, that is, granulosa cell development and maternal placenta development as well as intermediate mesoderm formation, regulation of cell growth involved in cardiac muscle cell development, positive regulation of striated muscle contraction, response to stimulus involved in regulation of muscle adaptation, intermediate mesoderm formation, voluntary musculoskeletal movement, growth plate cartilage development, extracellular matrix, and so forth. The HMM-specific temporal dynamic gene sets were further investigated for coexpressed gene sets and functional network modules through ebdbNet and GeneNet in R package and GeneMANIA [63–65] as shown in Figures 5 and 6.

### 3.6. Simulation Studies

We show that dynamic methods outperform approaches that do not explicitly address the time series nature of the data in simulation studies for validation and evaluation. We evaluated the performance of dynamic methods with simulation studies in which temporal features are already known as gold standard TDE (temporally differentially expressed) gene lists. Gold standard gene lists contain entire information to mimic RNA-seq time series profile if a gene is differentially expressed (DE) or equally expressed (EE) over time as reference set to compare to the results obtained from both dynamic and static methods in terms of recall and precision measurement. To this end, we generated simulated RNA-seq datasets with expression profiling data points representing nondifferentially expressed and differentially expressed genes in a series of time points by using different values of autocorrelation parameter (*φ*). We generated data for equally expressed genes by sampling time series process parameters (*w*) of a gene in invertible Gaussian ARIMA process with *φ* = 0. We generated data for differentially expressed genes across time points in the same procedures as *φ* = 0.1, 0.25, 0.5, 0.75, and 0.9, respectively. After time series process, regression effects and autocorrelation parameters were simulated for 1000 genes, 4 simulated datasets were generated by setting the varying number of time points and replicates in a time point, *nT* = 5 and 10, *nR* = 3, and 5 to compute *P* value, FDR, and credible interval of each gene for static and dynamic methods and compared to gold standards to obtain true discovery rates in our simulated datasets.

## 4. Conclusion and Discussion

**Figure 7:**
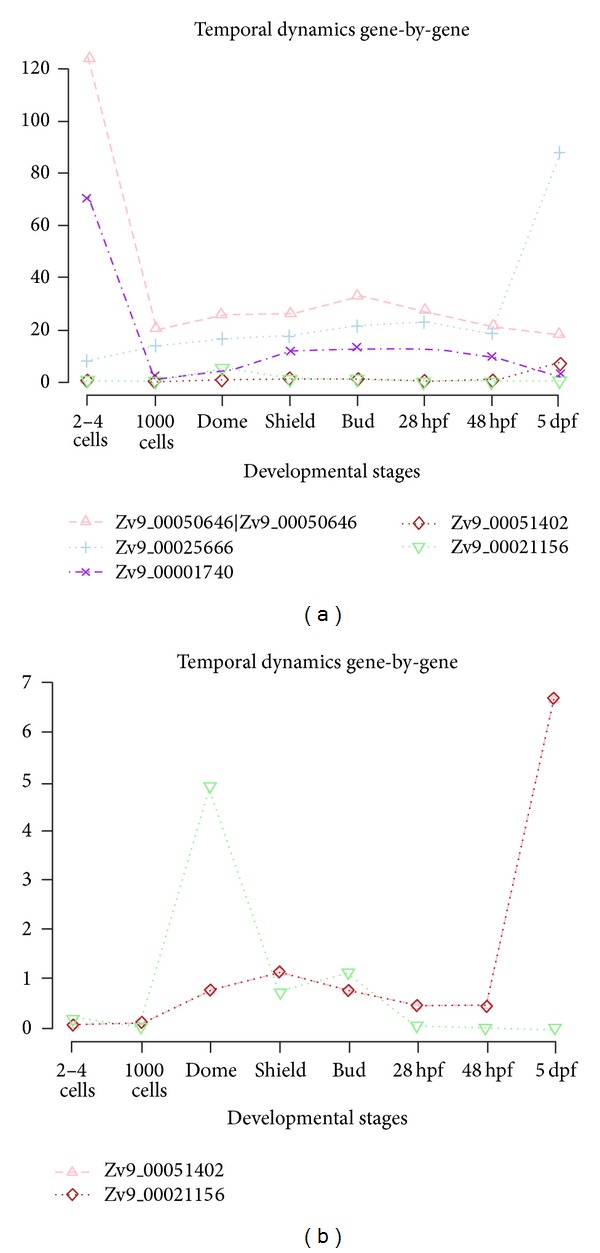
(a) Gene expression curves for five TDE genes in zebrafish data. Expression curves are presented during eight developmental stages comparing temporal patterns to each other in identifying statistical significant trajectories. (b) Two specific genes in gene-by-gene temporal dynamics with low expression levels via SETI in zebrafish data from (a). SETI enables identification of significant temporal patterns at low expression levels which are not detected by other existing static methods.

**Figure 8:**
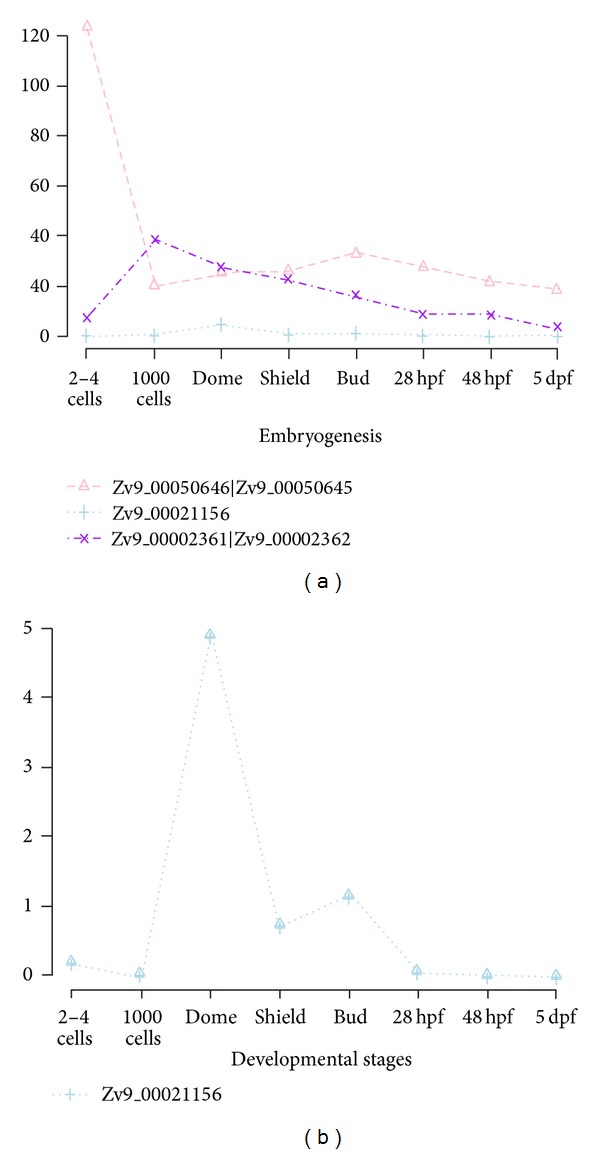
(a) A set of top cooperative TDE genes detected by Glass-d-score with FDR 0.05 as a candidate of cooperative gene pairs in coexpression in zebrafish data. Glass-d-score and corresponding FDR at cutoff 0.05 by resampling procedure under the null hypothesis that there are no TDE patterns across samples and those are shuffled with 1,000 repetitions. (b) One specific gene in coupled temporal dynamics with low expression level via SETI and Glass-d-score from (a). Glass-d-score is robust in identifying coexpressed genes over time at low expression levels.

We first performed pairwise comparisons using two simple static methods, Audic-Claverie statistics and Fisher's exact test. The congruent set of both of them is highly overlapped and we reported the results of Fisher's exact test as more common method in Figure 1. The dataset came from a sheep model with two different healing systems at four different days. This dataset provides an excellent design for identification of temporally and simultaneously differentially expressed (TDE) genes as we have two conditions at each time. This type of time course is referred to as factorial time course experimental design. The authors of [53] took a union set of all these combinations of pairwise comparisons in condition and time point to identify TDE genes. These approaches might provide insights and intuitively simple static methods are alternative in small experiments in general. Evidently, the methods for time series dynamics are still in their infancy. However, those algorithms all do not consider dependency between samples in time course and they assume that all samples are independently distributed, though sequential correlation is obviously observed in data as shown in Figure 2. We noticed that basically patterns of detection of temporal changes by static and dynamic method are different, albeit they agree in some degree. That is, most of temporal genes at low and moderate expression levels are detected as significant genes in dynamic methods, whereas, due to power issues of parametric static pooling methods and simplification of pairwise methods, static methods do a good job at high expression levels. To confirm robustness and reliability of gene detection methods in time series, a comprehensive comparison and evaluation with varying parameter settings closer to RNA-seq real world is further needed. At low and moderate levels, many genes which were not detected by static methods but dynamic methods still showed log2-scaled FC ~4 up to 5. We sought to test the ability of dynamic methods whether or not those identified dynamic-unique TDE genes are genuinely differential expression or just by a random chance because expressions have been more affected by noise at low and moderate levels in microarray, even though RNA-seq quality when compared to microarray has been much improved for now. In Figure 4, to assess temporally differentially expressed genes, we incorporated SETI with HMM algorithm in a sheep model within each condition to see a variety of time-varying trajectories since HMM provides only patterns of hidden latent variables (DE/EE) at developmental stages. Left panel shows three candidate genes at low and moderate levels and right panel shows another three candidate genes at high expression. To examine biological meanings in TDE genes detected by only HMM, we further performed gene clustering coexpression patterns to see if those gene sets have possibility of false negatives in altered expression of cooperative genes and gene functional pathway analyses. Notably, we confirmed meaningful biological functionalities and temporal patterns in dynamic specific TDE genes in downstream analyses, gene clustering, gene ontology, and pathway/network analysis. Interestingly, as 63 females were sampled in the data, some of gender-specific GO terminologies among significant ontology terms were identified, that is, granulosa cell development and maternal placenta development, intermediate mesoderm formation, regulation of cell growth involved in cardiac muscle cell development, positive regulation of striated muscle contraction, response to stimulus involved in regulation of muscle adaptation, intermediate mesoderm formation, voluntary musculoskeletal movement, growth plate cartilage development, extracellular matrix, and so forth. Consistently, HMM, SETI, and AR(1) model that account for time dependency Markovian property in the models identified more of statistically significant TDE genes than static methods regardless of expression levels. In summary, the approaches we described use a developed unified dynamic test framework that includes SETI with statistical significance testing, ranking temporal genes by AR(1) modeling and posterior probability of autocorrelation parameter, and HMM to classify temporal dynamic patterns. These methods seem to be robust regardless of the magnitude of expression (see Figures 7(b) and 8(b), and Supplementary Tables) and more sensitive than static methods as shown in Supplementary TDE Tables; moreover, TDE genes detected by dynamic specific methods were confirmed as temporal dynamics in clustering patterns and biologically significant modules in network analysis implying that the gene sets were not identified as false negative genes in static methods that samples over time are assumed independently. We anticipate that temporal RNA-seq experiments will be widely performed in the near future due to reduced sequencing cost and the rich information carried by these experiments. In this paper, we consider several statistical approaches that can explicitly model the time series nature in the data. We discussed the limitations of simple static pairwise comparison methods for time series data analysis; dynamic statistical framework for RNA-seq read count with statistical evolutionary trajectory index measure; autoregressive time-lagged AR(1) model; hidden Markov model; pairwise and multiple comparisons among trajectories to investigate coupled bivariate dependency between distinct SETIs; and pathway/network analysis in transcriptome data based on detected temporally differentially expressed genes. Thus, this study covers critical issues that have not been systematically addressed in temporal RNA-seq data and we hope this will motivate more rigorous developments of novel methods to model and analyze RNA-seq data. Of particular interest will be the extension of these methods to combined time series datasets from RNA-seq, proteomics, and metabolomics for *in silico* cell/organism predictive modeling [6]. In addition, it will facilitate cross-species comparative analyses of temporal gene expression to investigate developmental processes and disease progression such as aging and virus-mediated immune disease dynamics. Deep sequencing of mRNAs has been a popular and effective approach for quantification of alternative splicing events, and it is well known that more than 90 percent of human genes have multiple isoforms to produce different protein structures. Thus, an important future direction is also to extend the statistical framework of our dynamic methods to incorporate the characterization of isoform diversity in time course in detecting differential expression. 

## Supplementary Material

Supplementary Table 1: Temporally differentially expressed gene sets detected by statistical evolutionary trajectory index (SETI) where the Benjamini-Hochberg FDR is controlled at < 0.05 in maize data
Supplementary Table 2: Temporally differentially expressed gene sets detected by statistical evolutionary trajectory index (SETI) where the Benjamini-Hochberg FDR is controlled at < 0.05 in zebrafish data
Supplementary Table 3: Temporally differentially expressed gene sets detected by Poisson AR(1) model in maize data
Supplementary Table 4: Temporally differentially expressed gene sets detected by Poisson AR(1) model in zebrafish data

## Figures and Tables

**Table 1:** 2  × 2 contingency table.

	Reads from sample of type		
Tags in gene	Group A	Group B	
Gene g	*g*_+1,A_	*g*_+2,A_	*g*_.,A_
Not gene g	*g*_+1_ − *g*_+1,A_	*g*_+2_ − *g*_+2,A_	*g*_.,B_
Total	*g*_+1_	*g*_+2_	*g*_.._

**Table 2:** Statistical evolutionary trajectory index (SETI) of the top candidate genes where FDR is controlled at less than 0.05 in a sheep model data. The gene expression level is fitted on smooth spline function, autocorrelation of residuals is measured, and corresponding statistical significance is tested. In addition, trimmed mean of bootstrap and 95 percent of confidence interval are also provided in the table.

Top candidate genes	SETI (trimmed mean of bootstrap)	Bias of bootstrap	95% CI of SETI	*P*	FDR
A5D9H5	1.23 (1.23)	0.12	[1.02, 1.43]	0	0
A6QQB6	1.23 (1.27)	0.09	[1.00, 1.46]	0	0
A0JN96	1.23 (1.23)	0.09	[1.03, 1.43]	0	0
DUFFY	1.23 (1.23)	0.13	[1.02, 1.43]	0	0
A6QP68	1.23 (1.23)	0.10	[1.03, 1.42]	0	0
gi∣11992112	1.23 (1.23)	0.10	[1.05, 1.40]	0	0
